# Toward an Embodied, Embedded Predictive Processing Account

**DOI:** 10.3389/fpsyg.2021.543076

**Published:** 2021-01-29

**Authors:** Elmarie Venter

**Affiliations:** Institute for Philosophy II, Ruhr University Bochum, Bochum, Germany

**Keywords:** predictive processing, embodiment, mechanistic explanation, free energy, prediction error

## Abstract

In this paper, I argue for an embodied, embedded approach to predictive processing and thus align the framework with situated cognition. The recent popularity of theories conceiving of the brain as a predictive organ has given rise to two broad camps in the literature that I call *free energy enactivism* and *cognitivist predictive processing*. The two approaches vary in scope and methodology. The scope of *cognitivist predictive processing* is narrow and restricts cognition to brain processes and structures; it does not consider the body-beyond-brain and the environment as constituents of cognitive processes. *Free energy enactivism*, on the other hand, includes all self-organizing systems that minimize free energy (including non-living systems) and thus does not offer any unique explanations for more complex cognitive phenomena that are unique to human cognition. Furthermore, because of its strong commitment to the mind-life continuity thesis, it does not provide an explanation of what distinguishes more sophisticated cognitive systems from simple systems. The account that I develop in this paper rejects both of these radical extremes. Instead, I propose a compromise that highlights the necessary components of predictive processing by making use of a mechanistic methodology of explanation. The starting point of the argument in this paper is that despite the interchangeable use of the terms, prediction error minimization and the free energy principle are not identical. But this distinction does not need to disrupt the *status quo* of the literature if we consider an alternative approach: Embodied, Embedded Predictive Processing (EEPP). EEPP accommodates the free energy principle, as argued for by free energy enactivism, but it also allows for mental representations in its explanation of cognition. Furthermore, EEPP explains how prediction error minimization is realized but, unlike cognitivist PP, it allocates a constitutive role to the body in cognition. Despite highlighting concerns regarding cognitivist PP, I do not wish to discredit the role of the neural domain or representations as free energy enactivism does. Neural structures and processes undeniably contribute to the minimization of prediction error but the role of the body is equally important. On my account, prediction error minimization and free energy minimization are deeply dependent on the body of an agent, such that the body-beyond-brain plays a *constitutive* role in cognitive processing. I suggest that the body plays three constitutive roles in prediction error minimization: The body *regulates* cognitive activity, ensuring that cognition and action are intricately linked. The body acts as *distributor* in the sense that it carries some of the cognitive load by fulfilling the function of minimizing prediction error. Finally, the body serves to *constrain* the information that is processed by an agent. In fulfilling these three roles, the agent and environment enter into a bidirectional relation through influencing and modeling the structure of the other. This connects EEPP to the free energy principle because the whole embodied agent minimizes free energy in virtue of being a model of its econiche. This grants the body a constitutive role as part of the collection of mechanisms that minimize prediction error and free energy. The body can only fulfill its role when embedded in an environment, of which it is a model. In this sense, EEPP offers the most promising alternative to cognitivist predictive processing and free energy enactivism.

## Introduction

This paper defends an embodied approach to the predictive processing framework that is aligned with the broader setting of situated cognition. Inspired by principles in biological sciences and computer sciences, the predictive processing framework (henceforth, PP) has gained much popularity in cognitive science in recent years. This account of cognition turns the traditional account of cognition upside down: instead of the brain gathering information about the world, processing information, and then employing it in the output of action, the brain is constantly making predictions about the world. The account has been applied to explain a variety of processes in the brain, and aims to provide a unifying perspective of perception, action and cognition. This is agreed upon by most researchers in the field but the exact relationship between perception, action and cognition remains a contested topic in the literature on PP (Colombo and Wright, 2016). The surprising number of varied interpretations of PP may lead one to question whether they, in fact, refer to the same idea. The aim of this paper is to investigate this question and offer an embodied approach to PP (Clark, 2016; Kirchhoff, 2017, 2018; Kirchhoff and Kiverstein, 2019). I do this by differentiating between two popular interpretations—cognitivist PP and free energy enactivism—and then carving out an account most compatible with a strong embodied account of cognition. I take strong embodiment to mean that both neural structures and wider bodily structures *constitute* cognitive processes insofar as the body not only contributes to (or enables) the function of the predictive system (to minimize prediction error) but also directly fulfills this function without mediation by mental representations (Shapiro, 2004; Rowlands, 2010). This is contrasted with weaker embodiment claims which take cognitive processes to be *dependent* (to varying degrees) on bodily structures and processes (Rupert, 2009; Alsmith and de Vignemont, 2012).

The paper is organized as follows. I briefly describe the grounding principles of PP and focus on highlighting the distinction between the free energy principle and prediction error minimization^1^. The free energy principle sets out to explain all mind and life, and is typically applied to explaining why dynamic systems avoid disorder or dispersal (Friston, 2013a; Sims, 2016). Given the wide scope of the free energy principle, I set out to narrow down the discussion to the cognitive domain by presenting the relevant features of PP in terms of prediction error minimization. I then investigate two interpretations of PP: cognitivist PP leans toward a commitment to internalism, and free energy enactivism undertakes the task to explain dynamic, coupled engagement with the world. After critically examining the scope and explanatory ambitions of these two interpretations, I defend a mechanistic explanation of PP and use this as a starting point to develop an embodied account of PP. On the mechanistic approach, all components of the system that realize the function of the system are important and must be included in the explanation. Following this, I argue that the body be granted a strong constitutive role in an explanation of cognition because it fulfills the function of prediction error minimization without necessarily being mediated by mental representations.

## Setting the Scene

The objective of this section is to provide a bird’s eye view of the necessary features of predictive processing (PP). This section is intentionally vague given that more specific features will be discussed in the subsequent sections. What I wish to highlight is the distinction between the free energy principle and prediction error minimization. Though the two concepts are difficult to separate and often used interchangeably in the literature, they make different predictions and vary in scope and application (Bruineberg et al., 2018; Hohwy, 2020). Any description of PP starts with an understanding of the free energy principle which is defined as follows: “any self-organizing system that is at equilibrium with its environment must minimize free energy” (Friston, 2010) where free energy refers to a state associated with disorder or uncertainty. The principle is based on the fact that biological systems have a limited range of states in which they can survive. It is therefore necessary for an organism to maintain itself within its possible range of states by minimizing disorder and uncertainty; failure to do so leads to dispersal and ultimately death. The idea upon which PP is build is that in order for a system to maintain itself within a particular range of states, it requires the capacity to predict future states. In sophisticated systems, like human agents, this means tracking and representing the causes of sensory states. This process is realized by generative models with different sets of priors about the environment and the agent. The primary function of these generative models is to maintain a set of hypotheses about the world that generates the most accurate predictions of the incoming information and consequently minimize uncertainty about the environment (free energy). Free energy is evaluated using two factors: an agent’s sensory states and a recognition density (i.e., the aforementioned probabilistic representation of the hidden causes of sensory states) (Friston, 2010). Free energy minimization is a principle of optimization that can be applied at many different levels of analysis and at different timescales, explaining how we maintain bodily states such as, for example, blood sugar levels (Seth, 2013) to how we maintain an optimal narrative model of ourselves (Hohwy and Michael, 2017), and even explaining social cognition by means of interoceptive inference (Fotopoulou and Tsakiris, 2017). The use of “generative model” is cautiously applied and does not *necessarily* imply contentful representation because it can be applied beyond the neural domain. When the free energy principle is applied to the neural domain, the amount of free energy is calculated as the sum of all prediction error which is defined as the divergence between the probability distribution encoding the sensory states and the recognition density. It is interpreted as the mismatch between what is predicted and the incoming sensory stimuli.

In the neural domain, PP is defined by the idea that processing stimuli is driven by top-down processes. This is commonly referred to as prediction error minimization (Hohwy, 2013). To see a structured world is to use existing generative models of the world to shape a virtual version of sensory perturbations from the top down. Thus rather than reconstructing the world, the system is “constantly trying to *guess the present*” (Clark, 2017b p. 727, emphasis in original). Generative models are constantly updated so that the best possible top-down predictions are generated to meet bottom-up transmissions. Better top-down predictions mean that more incoming information is matched and explained away (which results in less uncertainty). The process of “explaining away” incoming information leaves only prediction error to be propagated within the system. This bidirectional process occurs at different spatial and temporal scales operating at many different levels of a processing hierarchy where, at each level, the system is trying to predict its own sensory states. The important feature in this schema is interaction between the different levels, where higher level predictions involve more abstract and temporally extended states and lower-level predictions process more fine-grained states, such as lines, edges and textures of surfaces. The predictions that pervasively determine perceptual experiences are extracted from higher levels and prior knowledge based on statistical estimation. Statistical estimation refers to a calculation of accuracy and precision within a range of likely and probable predictions that explain sensory causes. The function of the whole system is for top-down predictions to meet the incoming signals and become more successful at making predictions about the world. Estimates at each level in the hierarchy are also predictive of each other in order to assist with the successful execution of this function. Thus, prediction is not just from one level to the next but also occurs between models at a single level. This strategy is efficient in that it minimizes computing power because mismatches between top-down and bottom-up information only update generative models which already exist (Metzinger and Wiese, 2017).

Prediction error minimization is the main objective of the system (the brain is commonly the system referred to in this context). Predictions can be accurate or inaccurate to varying degrees. There is a direct correlation between the accuracy of a prediction and how well fitted a generative model is in that an accurate prediction is an indication of a successful generative model. If the prediction is accurate, nothing more needs to be done and the generative model is accurate with respect to the state of the world. If bottom-up signals are not accurately predicted, the mismatched information is transmitted as prediction error until the model (more or less) matches the state of the world. Prediction error can be minimized in two ways: perceptual inference and active inference. Perceptual inference involves model revision based on prediction errors. Prediction errors are transmitted up the hierarchy and the generative model is updated. Active inference is a process in which the agent acts upon, or changes, the world in order to bring about the state of the world predicted by the current best generative model. It can be argued that active inference can be explained in entirely internalist terms insofar as predictions about bodily movements and its causes on the environment is an inferential process. Cognitivist PP is committed to the view that active inference is a result of “the sensorimotor system passing predictions of proprioceptive input to the classic reflex arcs, which fulfill them and thereby cause action” (Hohwy, 2016, p. 262). I reject this view and will develop an account on which active inference is construed as direct (not inferentially mediated) engagement with the environment (Bruineberg et al., 2018; Kiverstein, 2018). On this view, perceptual and active inference are intricately linked rather than one being in the service of the other. Active inference captures the action-oriented nature of PP which enables predictive control and has the positive effect of enabling an agent to act in order to regulate vital parameters. Importantly, it is the aim of the system to exercise predictive control by deciding which strategy to use for successful prediction error minimization in the long run. If the system always adapts to signals regardless of how noisy and uncertain they are, it runs the risk of overfitting the generative models—making it unreliable as a way to structure the world. On the other hand, not adapting the models when prediction error is propagated upwards, runs the risk of underfitting the model. The need to explore the environment and seek sensory information then becomes redundant. It is therefore important for the system to strike a delicate balance between changing the model and its parameters, on the one hand, and maintaining the parameters and changing the incoming signals.

The features discussed in this section form the foundation for an understanding of PP in terms of prediction error minimization as it is derived from the free energy principle. These features are interpreted in various ways and are highlighted to various degrees. I discuss two interpretations of predictive processing before developing the EEPP account. The first interpretation, I call cognitivist PP. This account is spearheaded by Jakob Hohwy who refers to his account as prediction error minimization; it is also referred to as “conservative predictive processing” by Clark (2015). I refrain from using Hohwy’s terminology to avoid confusion given that my own account makes use of prediction error minimization as a function but does not restrict this function to the neural domain. On Clark’s terminology, my account would also be understood as conservative given that I do not propose to discard the notion of representation. But I grant a constitutive role to the body so I set my account apart from an internalist, cognitivist interpretation of PP. The second interpretation that I discuss arises from a combination of radically enactive cognition (REC) and “radical predictive processing” Clark (2015). I call this free energy enactivism to highlight the amplified role of the free energy principle in cognitive processing.

## Cognitivist Predictive Processing

The cognitivist interpretation of predictive processing builds on the features discussed above and construes the brain as a prediction error minimization system. Prediction errors signal the mismatches between bottom-up sensory signals and multi-area, top-down flows of *neuronal* activity (Clark, 2017b, p. 727, my emphasis) which serve to reconstruct the external reality. This process requires that the mind is an independent system that processes information entering from the outside world, reconstructing and mirroring the world for the agent to interact with. Anything that requires us to interact with it must be modeled. This distinction between mind and world enforces a strong and rigid evidentiary boundary between what happens in the external world and the generative models in the brain. In this sense, cognitive processes are inferentially secluded and neurocentrically skull bound (Hohwy, 2016, p. 259). Thus, any inputs beyond the sensory organs are outside the evidentiary boundary and can only be reconstructed (represented) in the brain. Hohwy (2016) epistemically decouples the brain from the body and world by suggesting that the brain, in implementing prediction error minimization, is self-evidencing. The brain has a model of the environment in which it is found and is continually updating generative models or changing input. It is equipped with the task of explaining away sensory input and, in doing so, it generates evidence for its own existence. This does not depict the brain as a passive organ; instead the brain is actively sampling evidence that matches its predictions and exploits the body as a tool in this undertaking. Perception is a process of representation only realized in the brain that infers distal information based on “partial and fragmentary information available in the sensory signal” (Clark, 2017b, p. 729). Our access to the world is bounded by prediction error minimization.

On this approach, action is explained in terms of proprioceptive prediction in that the approach construes action as a result of the brain’s predictions about what state the body should be in (Friston and Stephan (2007), Friston (2010), and Hohwy (2016)). Action is an inferential process that starts in the neural domain and then “the body as it were goes away and does its own thing until the predictions come true” (Hohwy, 2016, p. 276). On the cognitivist PP approach, having embodied access to the world is not a necessary condition of the prediction error minimization system—it just so happens that we have bodies and therefore action is more likely (Hohwy, 2018, p. 135). Thus, predictive control is not explained in terms of agentive access to the world, or coupling between agent and environment, but rather in terms of the brain selectively sampling the sensory evidence presented to it (Burr and Jones, 2016). The brain is in the spotlight and the body in itself plays no constitutive role because “the mind begins where sensory input is delivered through exteroceptive, proprioceptive, and interoceptive receptors and it ends where proprioceptive predictions are delivered, mainly in the spinal cord” (Hohwy, 2016, p. 276). On this view, the body is important only insofar as it is represented in the neural hierarchy. Neural populations transmit commands for action based on sensory input. There is no direct access and engagement with the real world.

A notable implication of cognitivist PP is that the mind can be explained in entirely “internalist, solipsistic terms, throwing away the body, the world, and other people” (Hohwy, 2016, p. 265). The scope of cognitivist PP is thus limited to the brain, and all other phenomena (including the body and tools in the environment) only serve as resources to fulfill the function of prediction error minimization. Prediction error can be minimized using two strategies: (1) changing sensory input through action or (2) changing the internal models of the world. On the cognitivist PP account, both these strategies are explained as occuring primarily within the bounds of the skull. All processes relating to the agent are cashed out in terms of what happens in the cortical hierarchy. Action is enslaved in service of the brain and parts of our own bodies that are not functionally sensory organs are not constituents of cognitive states (Hohwy, 2016, p. 269). Bodily movements, as well as processes such as heart rate, are all inferred processes, lying beyond the evidentiary boundary. Construing the body as just another cause in the environment implies that it is nothing special, and neither is representation thereof (Hohwy and Michael, 2017). Although bodily movement is understood as facilitating prediction error minimization, and thus still a key feature in the cognitivist PP account, the role of the body is largely underplayed. Bodily movement is understood as an inferential process that arises from reconstructing the world rather than as enabled by sensory co-ordination.

Cognitivist PP does not grant the body any constitutive role in cognition. This is a symptom of the account taking a functionalist approach to explanation and limiting the function of prediction error minimization to the brain. The primary function of the brain, on this approach, is to minimize prediction error and all other phenomena serve only as tools to fulfill this function and are not explanatorily valuable in themselves. Hohwy (2015) sees value in a functionalist explanation because, he proposes, it provides a unifying principle for understanding what the brain does. Perception, for example, is specified in terms of a particular function—generating the best possible model of what is observed—then broken down into further sub-capacities such as estimating precision and fitting statistical models. These sub-capacities are then organized in a way that realizes the overall function of the capacity to be explained. Consider a non-biological example of functional explanation: assembly line production (Cummins, 2000). In an assembly line, workers are assigned a task and the final product is successfully produced because each station has fulfilled their assigned function. The entire system can successfully fulfill its overall, unified function (producing a product) because each station fulfilled its given tasks in an organized way. An assembly line can be explained without making reference to the product being produced, the factory in which it is produced, and the number of stations involved in production. Similarly, a functional analysis limiting prediction error minimization to the brain does not make reference to the whole system that realizes the function but only to the function itself. Hohwy (2015, p. 17) acknowledges the problem of realization, and that a system has certain kinds of mechanisms that realize the function but limits talk of realization to neuronal circuitry. This approach is paradigmatic pure functionalism which is strongly committed to explaining only the functional role of a phenomenon and not how it is realized (Cummins, 2000; Egan, 2018). Although this can provide much insight into why the brain processes information in the way it does, and why we interact with the world in particular ways, the account leaves much desired in terms of explaining how prediction error minimization is realized. Providing an account of the “how” would require consideration of all components of the system including, I argue, the constitutive role of the body. In the next section, I discuss free energy enactivism which grants the body a central role in its explanations but at the cost of blurring the boundaries between what is understood as being cognitive and what is not.

## Free Energy Enactivism

The fundamentally active and world-involving nature of predictive processing (PP) offers a point of agreement with enactivism. But despite the central role of action for cognition in PP, a tension arises because the PP framework does not seem to be complete without appeal to generative models that require contentful representations. Radically enactive cognition (REC) suggests that basic (i.e., not mediated by or involving language) cognition is contentless and non-representational (Hutto and Myin, 2013, 2017) and since PP is grounded in the manipulation of representational contents, the two accounts are in tension. REC outright rejects the cognitivist interpretation of PP and even a more “radical” version of PP that posits action-oriented representations. REC’s objection is that any account that appeals to representations in its explanation must deal with the hard problem of content which involves explaining where the brain gets its conceptual resources from to represent information and make inferences (Hutto, 2018). According to REC, no acceptable answer has been offered by proponents of PP. Hutto (2018, p. 21) suggests that prediction error minimization can be explained in terms of embodied anticipations that are “grounded in structural and functional neural and other changes wrought through an organism’s history of interactions.” This implies that our actions and experiences change our neural setup not in terms of neural representations but rather in that the neural domain is “set up to be set off” (Prinz, 2004, p. 55). Thus, information processing is not the same as energy transfer or electrical activity in the brain but rather information-as-covariance (Hutto, 2018, p. 22). But the account offered by REC leaves much to be desired in that it does not provide a positive proposal about how else we could cash out the idea that the predictive system harbors generative models, that something or other is expected or predicted, and that there are matches or mismatches between top-down predictions and bottom-up signals.

Building on the same foundations as radical enactivism, another radical interpretation of PP has been developed in the literature; I call this interpretation free energy enactivism. Free energy enactivism, unlike cognitivist PP, proposes that the free energy principle and the inferential account of perception and cognition are conceptually independent (Bruineberg et al., 2018). The free energy enactivist approach maintains that the dynamic coupling between organism and world suffices to explain cognition and thus the notion of inference in the brain is not required. The premise for the free energy principle providing an account of cognition is that free energy is a function of sensory states and the internal dynamics of a biological system. This function is extended to the whole embodied organism, and not limited to reconstructing the structure of the environment in terms of representations. Instead, it is self-maintaining processes that endow an agent with a lived perspective and any disequilibrium shapes the way in which the world is perceived (Bruineberg et al., 2018, p. 2,426). Perception, on this view, is a result of the agent being open and responsive to affordances based on its metabolic and thermal disequilibria. Free energy enactivism thus understands perception as worthless without reference to action.

Free energy enactivism aims to provide an account that unifies biology and cognitive science. One of the radical claims put forward by free energy enactivism is that “the free energy principle applies not just to humans but to all living systems, including the simplest of life forms such as bacteria” (Bruineberg et al., 2018, p. 2,419). The principle has also been applied to plant cognition suggesting that plants predict the environmental factors that cause sensory stimulation (Calvo and Friston, 2017). Rather than appealing to the notion of representation, the generative models that predict the structure of the world has the function of mediating the organism’s interactions with the world rather than reconstructing them. How does free energy enactivism appeal to models of the world without the notion of representation? Friston (2011; 2013b) suggests that it is not the case that an agent merely reconstructs a model of the world, but the agent is a model: the organism *embodies* an optimal model of its environment. In this sense, environmental features play a constitutive role in cognition; the internal and external morphology of an agent is constrained by the environment in which it is found. This is a bidirectional process because an organism’s morphology also determines the environment in which the organism can survive. The interplay varies along timescales in that the agent may adapt to the environment in the long term but will change the environment for shorter term survival and efficiency.

Construed in this way, free energy enactivism illustrates a deep continuity between mind and life which is typical of enactive approaches to cognition. On this view, the free energy principle applies to bacteria and plants as much as it applies to human agents in that these living systems engage in adaptive behavior (Kirchhoff and Froese, 2017). There is an implication that follows from this. If minimizing free energy is sufficient for mind and life, then all systems that resist disorder (or stay within bounds) exhibit mentality and are alive. There are two ways such a claim can be supported. Either one holds the view that mentality is not limited to living systems or by maintaining that life and mind are ubiquitous features (Kirchhoff and Froese, 2017). Both options give rise to panpsychism unless something further is added to the equation. The worry is that the scope of free energy enactivism is too broad in application and seemingly applies to non-living, non-cognitive systems. In other words, the boundaries between living, cognitive systems and the external non-cognitive world are blurred.

Furthermore, the free energy principle is construed as a nomological principle that all living systems abide by. It has been described as “normative” (Friston, 2013a), an “overarching rationale” (Clark, 2013), and a “law-like regularity” (Hohwy, 2013). On the radical construal by free energy enactivism, an organism is dynamically coupled with the environment through generalized synchrony (Friston, 2013b). But the notion of generalized synchrony is observed even in pendulum clocks that eventually synchronize through the beams from which they are suspended. This implies that one clock infers the state of another and is a generative model of the dynamics of the environment (Bruineberg et al., 2018, p. 2,437). Taking the nomological explanation presumed by free energy enactivism seriously means that all instances of generalized synchrony are instances of free energy minimization. And free energy minimization is a sufficient condition for a system to be a living system. The implication is that by applying a general law such as the free energy principle to dynamical systems, from pendulum clocks to human cognition, the explanatory value of the principle is lost. “Laws simply tell us what happens; they do not tell us why or how” (Cummins, 2000, p. 119). Arguably, the free energy principle can explain the capacities of dynamical systems but it does not follow that it can predict all capacities of such systems (or similar systems)—despite its ambitions to do so. The free energy principle serves well to explain the organization of dynamical systems, but it does not follow that the principle then adequately explains cognition. The free energy principle is very wide in scope and overshoots by trying to fully explain cognition. Rather than ambitiously attempting to explain all phenomena with a single principle, the aim should be to search for an explanation that captures the regularities of whole embodied organisms and their interaction with the environment. The free energy principle is presented as doing exactly this but I argue that despite how it is presented, the explanandum of the free energy principle under free energy enactivism is not the same as that of PP.

One way to sidestep the challenges is to consider the differences between non-living dynamical systems, simple life-forms and complex human agents where the latter may employ representational knowledge structures. But free energy enactivism rejects this position and suggests that an appeal to representation is not necessary. My proposal is that only by explaining additional components of the sophisticated system do we get an explanatorily useful account of perception, action and cognition. The free energy enactivist interpretation of PP also leaves much to be desired in terms of accounting for all the components of the system that realize prediction error minimization. I address this gap in the rest of this paper.

## Finding a Third Way

Predictive processing (PP) is committed to providing causal and constitutive explanations of cognitive capacities. Achieving this requires investigating what kind of (methodological) explanation fits well with PP. I suggest that the explanatory methods of PP should be aligned with the mechanistic approach to explanation and that this requires including all components that realize cognition (including the body). Currently, both cognitivist PP and free energy enactivism offer no more than mere description and functional analysis, and though these accounts do not reject a mechanistic approach, they fail to include *all* components in their respective explanations. The two accounts that I have unpacked also differ in what they take to constitute cognition. Cognitivist PP restricts cognition to the neural organ and anything beyond that, including the body, only serves as a tool to minimize prediction error. The body as mechanism is reduced to how it contributes to prediction error minimization which is realized only in the neural domain. Free energy enactivism, on the other hand, extends cognition beyond the organism into the world such that the boundaries between cognitive and non-cognitive phenomena become blurred. I propose that these views represent extremes that alone do not successfully explain cognition. Instead, I defend a strongly embodied view that embeds the agent in the environment in which it is found (Friston, 2011; Pezzulo, 2014; Clark, 2015). I will argue that this is aligned with the mechanistic approach of explanation which identifies all relevant components of a system in realizing the phenomenon to be explained thus respecting both functional and structural properties. Jakob Hohwy, the key proponent of cognitivist PP, identifies the mechanistic potential of the framework but remains committed to a functionalist explanation of cognitive capacities in virtue of concepts such as precision, prediction error and model optimization (Harkness, 2015, p. 6). I suggest that PP can explain common sets of sub-capacities of cognition and their organization. This can then be used to provide an account of how cognitive capacities are realized in different biological systems. On the strong embodied view, the body is a constituent of cognition, i.e., it is part of the mechanisms that realize the function of the system. An account of PP should include an explanation that includes the body as realizer of prediction error minimization given that all components of the system and their capacities must be explained on the mechanistic view.

Explanation in cognitivist PP and free energy enactivism is aimed at describing free energy minimization (or prediction error minimization), but both accounts neglect to consider the structures and mechanisms that realize this phenomenon. This is not to say that the contributions of these accounts are in vain but functional explanations can be enriched with mechanistic explanations (Piccinini and Craver, 2011; Harkness, 2015). Functional explanations often serve as first steps in mechanistic explanation in the sense that functional explanations provide sketches of mechanisms and the gaps are then later filled out (Piccinini and Craver, 2011, p. 284). Mechanistic explanations identify the relevant components of the system and respects the importance of both functional and structural properties. Given the importance of the structure of the system in which a phenomenon is realized, I suggest that mechanistic explanation serves PP better. Adopting a mechanistic approach enables the explanation of the capacities of the system and its component parts as opposed to only explaining the functions and effects of the system. The structures and processes that realize prediction error minimization are explained rather than merely describing it via functional analyses or nomological principles.

### Mechanistic Explanation and Predictive Processing

Mechanistic explanation involves identifying the relevant parts of the mechanism, determining the operation they perform, and providing an account of how parts and operations are organized such that, under specific contextual conditions, the mechanism realizes the phenomenon of interest (Bechtel, 2009, p. 553). The Watt governor can serve as an example here and is often used as an analogy in dynamical systems theory of cognition. A Watt governor has the function of regulating the speed of engines. It functions to keep a system within a particular state (or range of states) and is constituted by several independent parts: the flywheel, the spindle and arms, and a type of linkage system connected to a valve. Each component of the governor operates on its own principles and performs a specific operation which contributes to the overall function of the system. It is because the spindle arms fulfill their function of rising and falling in response to the speed of the flywheel that their angle can be used to manipulate the linkage system. The spindle arms then open or close the valve allowing more or less fuel to pass through, increasing or decreasing the speed of the engine. The valve has no access to the speed of the flywheel without the spindle arms and linkage mechanism. All these mechanism form part of the system because they “encode” information that can be used by the valve. The Watt governor is a control system which is dependent on feedback to revise and redirect the behavior of parts of the mechanism.

There are uncanny similarities between the Watt governor as a mechanism that keeps the speed of engines within a particular range of states and the prediction error minimization system that functions to keep a living organism within a particular range of states. I suggest that the cognitive system comprises the whole embodied agent which includes the nervous system, the body, and relevant aspects of the environment. Like the Watt governor, each component of the embodied agent is a mechanism which operates on its own principles and performs specific operations, together contributing to the overall function of minimizing prediction error and keeping the agent within a particular range of states. The whole embodied agent is a control system and relies on feedback to control and direct motor activity and behavior. I suggest that prediction error minimization is not *only* performed through an interplay between predictions in the brain and activity at the sensory boundary (as proposed by cognitivist PP). Instead, we should think of prediction error minimization as the result of each component of the system (including the body) operating on its own principles and performing its own functions. For example, the body, in virtue of being a model of the environment, minimizes free energy by adapting accordingly across a long-term timescale. Prediction error minimization is realized by generative models in the brain and together with bodily movements the function is fulfilled. Representational mental states constitute only one component of the overall mechanistic system. The body is also a constitutive component in the process of minimizing prediction error and should not be treated as only a tool to fulfill the function of the brain. Each of the components of the system fulfills its own operations allowing the system to use the information to minimize prediction error in the long run.

## Embodied, Embedded Predictive Processing

As I have unpacked in earlier sections, prediction error minimization and the free energy principle are not identical concepts. They differ in scope and explanation. This view has recently been argued for by Hohwy (2020) who proposes that the free energy minimization account provides a conceptual and mathematical analysis that is primarily a nomological explanation and PP offers a falsifiable process-theory that is a mere application of the free energy principle. Another approach that separates free energy minimization and prediction error minimization is offered by Bruineberg et al. (2018) who propose that perceptual inference is not compatible with the claims made by the free energy principle. Analyzing these two arguments lies beyond the scope of this paper but is worth mentioning as key players in the debate separating the two concepts. The account that I develop is based on the separation of prediction error minimization and the free energy principle. Yet it cannot be neatly separated from either, and it does not need to be because it does not reject the compatibility of the two concepts. EEPP fits into the larger ambitions of the free energy principle and is also a way of explaining how prediction error minimization is realized. In this sense, the account that I develop is more sympathetic to that of Hohwy (2020) as opposed to that of Bruineberg et al. (2018) because my account does not commit to the idea that free energy minimization and perceptual inference are incompatible. Rather, these processes are realized at different levels of the cognitive system and the different mechanisms of the system operate on their own principles. Making space for both these concepts in a single account provides at least one good reason to consider EEPP as a viable alternative approach. Prediction error minimization is a process that gives rise to perception and (to a certain degree) enables action. Free energy minimization is implemented at the level of the whole embodied organism in virtue of the agent being a model of the environment. The embodied agent is embedded in the environment and engages in active inference to minimize uncertainty and disorder in the long term. Some insight about cognition can be derived *a priori* from the free energy principle, but the principle alone is too wide in scope to tell us all we want to know about cognition. Cognitivist PP, on the other hand, makes use of the free energy principle to develop an account of prediction error minimization but consequently restricts the scope of explanation to the neural domain underplaying the role of the body. The account that I develop will show that both these approaches contribute useful insights to our understanding of cognition but that by continuing to develop in opposing directions, the debate is losing sight of the phenomena in question: cognitive, embodied agents embedded in the world.

As cognitive, embodied agents we are directed at the world in a structured way. This capacity and our ability to act on the world is what sets us apart from other non-living systems. As I will argue in the next sections, the mechanisms that constitute cognition are not restricted to the neural domain. Prediction error minimization is instantiated not only by the neural domain but involves the whole system comprising the nervous system, body and relevant aspects of the environment (Anderson, 2014; Pezzulo, 2014; Clark, 2017a). I propose that prediction error minimization is deeply dependent on the body of an agent, such that the body-beyond-brain plays a constitutive role in cognitive processing. The body plays three constitutive roles in cognition:

1. The body *regulates* cognitive activity, ensuring that cognition and action are intricately linked. A prime example of this is the outfielder’s problem.
2. The body acts as *distributor* in the sense that it carries some of the cognitive load of neural structures. This is illustrated by examples such as interoception and the use of gestures.
3. The body serves to *constrain* the information that is processed by an agent. This is supported by the idea that the agent is a model of the environment.

The descriptions of these roles are not separable in a very clear way and often a single example can be used to explain multiple roles. I unpack each of these roles in the following sections.

### The Body as Regulator

The idea that cognitive processes serve to accommodate interaction with the world as opposed to reconstructing the world fits well to our understanding of the body as regulator. In embodied cognition approaches, the body as regulator thesis states that “an agent’s body functions to regulate cognitive activity over space and time, ensuring that cognition and action are tightly coordinated” (Wilson and Foglia, 2017). The embodied PP account explains how agents are geared toward fast, successful, and fluent engagement with the environment, using simple routines and minimal representation. The whole embodied agent includes a cognitive system that is made up of several mechanisms each operating on its own principles of operation. The body serves as regulator insofar as it enables the agent to perceive and interact with the world through embodying the causal structure of the dynamics of the environment and itself. Successful movement and action in the world are possible because of coupling between agent and environment and does not necessarily require reconstructing the sensory signals. Consider the outfielder’s problem: this scenario would involve a series of complex, action-sensitive information streams being fed to the brain—as if the agent is actually running to cancel the optical accelerations of the ball (Clark, 2017b, p. 735). The complexity involved in such a process would seem to count in favor of an account that can explain action and inference in simpler, embodied terms. This captures the notion of ecological efficiency which calls for a division of labor between brain, body, and environment. Division of labor between mind, body, and world enables the “productively lazy brain to do as little as possible while solving (or rather, while the whole, environmentally-located system) solves the problem” (Clark, 2015, p. 12). The cognitivist PP account can deliver an explanation of the outfielder’s problem but not without “throwing away” the world and the body. For the cognitivist, the action-perception process involved in the outfielder’s problem is one of inference that is a result of generative models that reconstruct a mirror of the world. The function of the system, on this account, is to generate hypotheses and find the best explanation of the sensory perturbations (the ball is moving and will drop to point x so in order to minimize prediction error, the outfielder must predict where the ball will land and then act in the world to move to point x). But on the embodied account that I develop, the function of the predictive system is to accommodate sensory perturbations to enable action in the world (the outfielder moves their body in such a way as to stay in a particular angle to the ball until meeting at the same point).

The embodied system is efficient because it uses minimal resources to capture what is necessary to act in the world. Navigating my way through a busy street is a complex task that requires movement of the body, adapting to uneven sidewalks, avoiding running children and other obstacles. The body regulates the agent’s interaction with the environment in virtue of the coupled dynamics between the environment and the body. This means commanding models with the least prediction error or with the least sensory signal to “explain away.” This notion requires an evidentiary boundary to distinguish between inferences and what is predicted. Cognitivist PP takes this boundary to be solid and clear “…with the brain on one side and the worldly and bodily causes on the other side” (Hohwy, 2016, p. 281). But on the embodied approach, the boundary becomes flexible and immutable (Clark, 2017c; Kirchhoff and Kiverstein, 2019). This does not mean the boundary does not exist—this would lead to the dissolution of the predictive task^2^. Instead, the boundary is determined by the agent and her *lived* body. It is not necessary for the body of the agent to be modeled and predicted in the same way as the external world because it does not lie outside the boundary. The boundary is determined by the physical lived body of the agent insofar as the agent embodies the causal structure of the environment which gives rise to a state of action readiness; the embodied agent is ready to act on the salient action possibilities in the environment. As active systems, we are constantly seeking which sensory input to sample next instead of passively matching prior probabilities with states of the environment. The body is crucial to the successful execution of this task because without it, there would be no interaction in the world, nor would there be any prediction error to minimize. The embodied PP account claims that the brain minimizes prediction error to accommodate the sensory barrage. Accommodating the sensory barrage involves other low-cost methods that do not imply action-neutral modeling of the environment.

### The Body as Distributor

The explanation above fits well with another way in which the body plays a constitutive role in cognition: as distributor. The body as distributor thesis states that “an agent’s body functions to distribute computational and representational load between neural and non-neural structures” (Wilson and Foglia, 2017). In the PP account, this means that prediction error is minimized by both neural and non-neural structures, such as the body-beyond-brain. A similar view is also put forward by Bruineberg et al. (2018) who propose that the predictive neural system does not “know” about the viable states in which the agent must maintain its body (a certain temperature, for example) and therefore such an embodied state can only be maintained by the body itself, i.e., without neural mediation. They call this embodied surprisal and use it as a premise to argue for the incompatibility of the free energy principle and prediction error minimization. Although I agree that the body can realize the function of prediction error minimization without neural mediation, I do not propose that these processes are separate and incompatible but rather that prediction error minimization in the neural domain and the minimization of, so-called, embodied surprisal are intricately linked.

On my account, action can be described as a process of inference that uses a non-reconstructive strategy to keep certain sensory stimulations within bounds. It is thus not necessary to reconstruct a model of the real world to plan, reason and guide successful behavior and action. Instead interaction with the environment is “a kind ofperceptually-maintained motor-informational grip on the world: a low-cost perception-action routine that retrieves the right information just-in-time for use” (Clark, 2017b, p. 737). The idea of body as distributor can be explored in EEPP by looking at how interoceptive information is processed. Perception of the body plays an important role in how we represent the world. For example, imagine you are watching a horror movie. As a result, your attention increases and your heartbeat accelerates. You hear a sound just outside the window which can be caused by several things. For the purpose of this example, let us limit the pool of hypotheses to two: (1) the wind is blowing a tree branch against the window, or (2) a thief is trying to gain entry into your house. Let us suppose you live in a low-crime area and have never experienced a break-in. The hypothesis with the highest prior probability should be that the wind is blowing a tree branch against the window. But given the interoceptive information and physiological state of your body, the thief-hypothesis has higher prior-probability^3^. This is because all the evidence (including interoceptive information) has to be explained. All available sensory information makes up the evidence against which a hypothesis is tested. Importantly, this sensory information is not limited to seeing hearing, smelling, tasting, and touch but also includes kinesthetic, proprioceptive and interoceptive information. In order to most effectively reduce prediction error, the whole embodied agent is involved. The body of the agent (in the above case, through interoception) contributes to the minimization of prediction error because it carries useful and reliable information.

### The Body as Constraint

The body as constraint thesis states that: “an agent’s body functions to significantly constrain the nature and content of representations processed by that agent’s cognitive system” (Wilson and Foglia, 2017). On PP, this can be understood in terms of how the agent models the environment. There are two ways in which the embodied agent models the environment. First, in terms of embodying a model of the environment, i.e., being a model of the environment. Second, in terms of generating action-oriented models of the world, i.e., having a model of the environment. Explaining in detail the two ways in which an agent models the environment in virtue of being an embodied agent requires more space than the scope of this paper allows and so the exposition that follows is brief. First, the embodied agent is not only modeled in the predictive system as part of the outside world but also acts as the point of reference from which the world is perceived. Interaction with the environment is made possible not only because the agent generates models of the world but the agent is its own best possible model of the world (Bruineberg et al., 2018, p. 2,425). The agent embodies a model of the environment in virtue of the coupled relation of internal and external dynamics, i.e., the structure of the environment is reflected in the embodied agent. In this sense, the environment and the embodied agent structure and constrain one another (Bruineberg et al., 2018, p. 2,422).

Second, the models that are generated in the predictive system are constrained by the structure of the embodied agent. Representing the world involves representing properties of objects such as shape, color, size and location but possibilities for action are also modeled and these affordances are only modeled as they are relevant and salient to the embodied agent. The affordance of sitting on a chair is only available to me, a human agent, insofar as I have the necessary limbs and joints that make this possible. My body thus constrains the models of the world that are generated; if I am paraplegic, a chair does not afford sitting but is rather an obstacle that I must avoid while moving around in my wheelchair. Most compatible with the embodied PP account is the notion of action-oriented representations. Action-oriented representations are aimed at driving specific action and are not reconstructive and detached from the world, nor are they disembodied (i.e., independent from the agent and their abilities). Action-oriented representations encode the affordances of objects as they are relevant and salient to the agent. Part of the predictive task is to anticipate and discriminate between things in the environment that matter to an agent and those that do not. In this sense, “the brain is constantly computing—partially and in parallel—a large set of possible actions” (Clark, 2016, p. 180). Concretely, this implies that the generative models in the predictive system are not detached and neutral reconstructions of the world but rather generative models of the possible ways in which the agent can interact with the world as constrained by their body. Such action-oriented generative models enable fluent interaction with the world because they are generated from the perspective of the agent, i.e., it is specifically relevant and salient to them in virtue of being an embodied agent, embedded in a specific environment.

## Conclusion

In this paper, I distinguished between two radical interpretations of the predictive processing framework. The divergence between the two positions is motivated by the conceptual distinction between the free energy principle and inferential perception (realized as prediction error minimization). As an alternative position, I propose a strongly embodied interpretation of predictive processing that take the whole embodied agent as well as relevant aspects of the environment to realize prediction error minimization. This alternative position includes the body as a constitutive part of cognition and as realizer of prediction error minimization. It also includes relevant aspects of the environment to constitute prediction error^4^ minimization. This can be understood in terms of affordances. Rather than include the whole environmental system in cognitive function (as proposed by free energy enactivism), I propose that only the brain and body-beyond-brain form part of the cognitive system. This implies that the boundary between cognitive and non-cognitive phenomena is not rigid and pre-determined but rather flexible and immutable. Developing a full account of EEPP is an enormous undertaking and requires contributions from many fields of science and philosophy. This paper aimed to deliver a starting point for such developments in the field rather than develop a fully fleshed out account.

## Data Availability Statement

The original contributions presented in the study are included in the article/supplementary material, further inquiries can be directed to the corresponding author/s.

## Author Contributions

The author confirms being the sole contributor of this work and has approved it for publication.

## Conflict of Interest

The author declares that the research was conducted in the absence of any commercial or financial relationships that could be construed as a potential conflict of interest.

## Footnotes

1 The divorce of the free energy principle from predictive processing is becoming more popular and several recent papers argue for such a separation. Hohwy (2020), for example, argues that the free energy principle offers a normative theory that is a mathematical and conceptual analysis whereas predictive processing is a falsifiable process-theory. Bruineberg et al. (2018) also argue for a conceptual distinction between free energy minimization and prediction error minimization. Although they also propose that the two concepts are incompatible whereas Hohwy and myself do not.

2 The debate on the nature and how far out the boundary extends beyond the neural domain is still hotly debated. Kirchhoff and Kiverstein (2019) defend an extended mind view and propose that the boundary (demarcated by the Markov blanket) extends all the way out into the world. On my account, the boundary is determined by the lived body of the agent. Concretely, this means beyond the neural domain to include the body but not including tools and other resources out in the world.

3 One could argue that another way of thinking about this is in terms of cognitive bias, for example if you were primed into expecting a thief because this a break-in occurred in the film you were watching. But this explanation does not suffice because interoceptive information is often more reliable than sensory stimuli.

4 There is a general divide between the action-first approach—construing affordances as byproducts of action plans—and the spectator-first approach—which highlights the role of belief-like representations of scenes with which an agent does not necessarily interact (Siegel, 2014, p. 51). I defend the view that affordances are relations between aspects of the environment and the abilities of an agent. This is in line with free energy enactivism which also maintains that affordances stand out as relevant in a specific situation lived by the agent and constitute the (pre-reflective) experiential equivalent of bodily action readiness: “the readiness of the affordance-related ability” (Bruineberg and Rietveld, 2014, p. 2).
